# Urinary incontinence and health-related quality of life among older Americans with and without cancer: a cross-sectional study

**DOI:** 10.1186/1471-2407-13-377

**Published:** 2013-08-07

**Authors:** Alexandra J White, Bryce B Reeve, Ronald C Chen, Angela M Stover, Debra E Irwin

**Affiliations:** 1 Departments of Epidemiology, University of North Carolina, Chapel Hill, NC, USA; 2 Lineberger Comprehensive Cancer Center, University of North Carolina, Chapel Hill, NC, USA; 3 Health Policy and Management, University of North Carolina, Chapel Hill, NC, USA; 4 Radiation Oncology, University of North Carolina, Chapel Hill, NC, USA; 5 Health Behavior, University of North Carolina, Chapel Hill, NC, USA

## Abstract

**Background:**

Few studies have investigated the impact of urinary incontinence (UI) on health-related quality of life (HRQOL) among cancer survivors. UI is prevalent in the general population and can be both an indicator of cancer and a side effect of cancer treatment. UI and cancer diagnoses have been associated with decreases in HRQOL. This study evaluates the prevalence of UI and the impact on HRQOL among older cancer survivors.

**Methods:**

The prevalence of UI among cancer survivors (breast, prostate, bladder, colorectal, lung, and endometrial/uterine cancers) and those without cancer was estimated using the SEER-MHOS database. Factors associated with UI were investigated using logistic regression and the impact of UI on SF-36 scores was determined using linear regression.

**Results:**

Over 36% of SEER-MHOS beneficiaries without cancer reported UI and higher prevalence was noted among cancer survivors (37%-54% depending on cancer type). History of bladder, breast, endometrial/uterine, or prostate cancer was associated with higher prevalence of UI. UI was independently associated with both lower physical component scores (PCS) (−1.27; 95%CI:-1.34,-1.20) and mental component scores (MCS) (−1.75; 95%CI −1.83, -1.68). A suggested decreasing trend in the prevalence of UI was associated with a longer time since cancer diagnosis.

**Conclusions:**

UI was highly prevalent, especially in bladder, endometrial/uterine, and prostate cancer survivors. Improved recognition of UI risk among cancer survivors will help clinicians better anticipate and mediate the effect of UI on individuals’ HRQOL.

## Background

Longer life spans will contribute to a large-scale population age shift in the United States over the next two decades. By the year 2030, there will be 71 million Americans over the age of 65 years, equivalent to approximately 20% of the U.S. population [1]. Increasing age is a known risk factor for many cancers, with more than 60% of new cancers and 70% of cancer deaths occurring in adults over the age of 65 years [2]. Older age is also associated with comorbid health problems and negative impacts on health-related quality of life (HRQOL) [3,4].

In the U.S., the prevalence of urinary incontinence (UI) or symptoms consistent with UI, is approximately 17% among men and 38% among women 60 years of age and older [5-7]. UI impacts many facets of an individual’s life, including work productivity and social, physical, psychological, and sexual health [8-11].

In addition, urinary symptoms can be caused by certain cancers (e.g., bladder, prostate, and gynecological cancers) and their treatments (e.g., prostatectomy or hysterectomy) [12-18]. Very little data exist on the prevalence of UI and its impact on HRQOL among cancer survivors, especially in elderly populations.

The current study’s main objectives are to evaluate the prevalence of UI among Medicare beneficiaries with and without cancer; to determine factors associated with UI including cancer type (prostate, breast, colorectal, endometrial/uterine, bladder or lung), demographic, and comorbid factors; and to investigate the impact of UI on HRQOL.

## Methods

### SEER-MHOS data linkage

The SEER-MHOS database links two population-based sources of data that provide detailed information about Medicare beneficiaries with cancer over age 65 years (the Surveillance, Epidemiology and End Results [SEER] program of cancer registries and the Medicare Health Outcomes Survey [MHOS]). Details of the SEER-MHOS data linkage have been published previously [19]. Briefly, the MHOS were designed to measure and track HRQOL outcomes of care provided by health maintenance organizations to Medicare Advantage Organization (MAO) enrollees. Every year, 1,000 randomly selected Medicare beneficiaries from each managed care plan under contract with the Centers for Medicare & Medicaid Services (CMS) are administered the MHOS. Participants are invited to complete both a baseline survey and a follow-up survey 2years later if they remain in the same managed care plan. The MHOS database includes self-reported socioeconomic, demographic, co-morbidity, race/ethnicity, health status, and functional status variables.

The SEER Program of the National Cancer Institute collects and publishes cancer incidence and survival data from population-based cancer registries covering approximately 28% of the U.S. population [20]. The SEER Program registries routinely collect data on patient demographics, primary tumor site, tumor morphology and stage at diagnosis, first course of treatment, and follow-up for vital status.

The SEER-MHOS linked data are considered by Health Insurance Portability and Accountability Act of 1996 requirements as a limited data set, requiring the investigators to sign a data use agreement before receiving the data. This exception allows for the release of deidentified SEER-MHOS data without obtaining authorization from individual patients (see *Federal Register*, August 14, 2002, page 53,235). IRB exemption was obtained from University of North Carolina at Chapel Hill.

### Cohort and participant selection

More detailed UI questions were included on the MHOS in 2003 and in later years. Thus, the current cross-sectional analysis includes both baseline and follow-up data for five SEER-MHOS cohorts: 2001 and 2003, 2002 and 2004, 2003 and 2005, 2004 and 2006, 2005 and 2007. For individuals with cancer and two completed SEER-MHOS surveys, the first survey after the most recent cancer diagnosis was selected for analysis. For individuals in MHOS without cancer, the first survey with the more detailed UI questions answered was selected. Individuals were excluded who (1) completed only one survey and did not answer the UI question (n=276 for those with cancer diagnosis, n=6,084 for those without a cancer diagnosis) or (2) had multiple surveys but the UI question was consistently missing (n=74 for those with cancer diagnosis, n=1,130 for those without cancer diagnosis). Men with breast cancer were also removed from the analysis (n=8) as there were too few to produce reliable prevalence estimates. Among women with gynecological cancer, we included only those with endometrial and uterine diagnoses as other gynecological cancer sample sizes were also too small to produces reliable estimates. Medicare beneficiaries who were classified as never diagnosed with cancer did not match to records in SEER and also responded negatively to the following question on the MHOS questionnaire: ‘Has a doctor ever told you that you had any cancer (other than skin cancer)?’ Records were ascertained for 6 different cancer types including (1) prostate (n=3,258), (2) breast (n=2,828), (3) colorectal (n=1,739), (4) endometrial and uterine (n=562), (5) bladder (n=749), and (6) lung (n=662), and for participants who had never been diagnosed with cancer (n=319,734).

### Measures

UI was defined by an affirmative to the following question: “Many people experience problems with urinary incontinence, the leakage of urine. In the previous six months, have you accidentally leaked urine? (yes/no)”. Further investigation of UI symptom bother was addressed in the following questions: (1)”How much of a problem, if any, was the urine leakage for you? (big problem/small problem/not a problem)”; (2)”Have you talked to your current doctor or other health provider about your urine leakage problem? (yes/no)”; and (3) “There are many ways to treat urinary incontinence including bladder training, exercises, medication and surgery. Have you received these or any other treatments for your current urine leakage problem? (yes/no).”

HRQOL scores were assessed by the Short-Form 36 (SF-36, version 1) and the Veterans Rand-12 (VR-12), which are scored on a T-score metric with higher scores reflecting better health. The MHOS moved from using the SF-36 to the VR-12 in 2006, consequently affecting the surveys from the last two cohorts. SF-36 and VR-12 physical and mental component scores have been rescored, using a published algorthim, so scores are equivalent [21]. The T-score metric was normed so that the average in the U.S. population is 50 with a standard deviation of 10 [22]. Two summary scores of the SF-36, Physical Component Summary (PCS) and Mental Component Summary (MCS), were used in this study.

Key covariates in this analysis included: smoking status, age at survey, sex, race, marital status, education, comorbid conditions, and difficulty with activities of daily living (which was defined as responding affirmatively to one or more of the following: difficulty getting out of a chair, using a toilet, walking, dressing, eating, bathing).

### Statistical analysis

Univariate distributions of demographic and clinical covariates were determined, including: urinary incontinence, smoking status, age at survey, sex, race, marital status, education, comorbid conditions, difficulty completing daily activities and time since cancer diagnosis to time of survey (categorized as: survey within 2 years after diagnosis, survey within 2-5 years after diagnosis, and survey >5years after cancer diagnosis). These time periods were selected to approximately represent active or early post treatment, short-term survival, and long-term cancer survival periods, respectively. A chi-square test was used to test the difference in the distribution of MHOS covariates between each cancer group and the non-cancer group. For HRQOL scores, a t-test was used. A variable for time since diagnosis was not tested for significance as there was not a clear referent group for which to compare it. The prevalence of individuals’ UI was calculated for each cancer type and for participants without cancer. The prevalence of UI was also investigated by whether or not the patient reported being bothered by their UI symptoms or sought treatment for UI. For all analyses, only those without missing data for the listed covariates were included in the models.

Logistic regression models were used to examine factors associated with UI by calculating Prevalence Odds Ratios (PORs) and 95% Confidence Intervals (95% CIs). These factors in the model included all cancer types (bladder, breast, colorectal, endometrial/uterine, lung, and prostate relative to no cancer group). Other variables were defined (index/referent) as follows: age at time of survey (>75, ≤75), smoking status (yes/no), race (other/non-Hispanic white), gender (male/female), marital status (other/married), education (> high school, ≤ high school), high blood pressure (yes/no), stroke (yes/no), chronic lung disease (including COPD asthma and emphysema) (yes/no), gastrointestinal (including Crohn’s disease, ulcerative colitis, and inflammatory bowel disease) (yes/no), diabetes (yes/no), difficulty completing one or more activities (getting out of a chair, using the toilet, walking, bathing, dressing, eating) (yes/no), joint pain (yes/no), and any cardiovascular disease (one or more of chronic heart failure, myocardial infarction, angina or coronary artery disease, or other heart condition) (yes/no). A second logistic model was performed among cancer survivors (excluding non-cancer individuals) and included the covariate “time since cancer diagnosis.” Cancer survivors with colorectal cancer were chosen as the referent group in this model due to a similar prevalence of UI compared to the no cancer group and sample size considerations.

Linear regression models were used to estimate the adjusted means on the SF-36 subscales and PCS and MCS scores, by cancer site and urinary symptom status, adjusting for relevant covariates listed above for the logistic regression models.

All statistical analysis was completed using SAS 9.2 (Cary, NC).

## Results

The current analysis utilizes data from SEER-MHOS cohorts who completed surveys between 2001 and 2007. Individuals were excluded (n=276 for those with cancer diagnosis, n=6,084 for those without a cancer diagnosis) if they completed only one survey and did not answer the UI question or had multiple surveys where the UI question was always missing (n=74 for those with cancer diagnosis, n=1,130 for those without cancer diagnosis). Men with breast cancer were also removed (n=8). After exclusions the final sample sizes for each of the 6 different cancer types were (1) prostate (n=3,258), (2) breast (n=2,828), (3) colorectal (n=1,739), (4) endometrial and uterine (n=562), (5) bladder (n=749), and (6) lung (n=662), and for those who had never been diagnosed with cancer (n=319,734).

The distributions of demographic and clinical covariates by cancer type are shown in Table 1. Only 46.1% of individuals without cancer were over 75 years of age at time of survey compared with the cancer groups, which ranged from 51.1% (lung) to 64.9% (bladder). In general, the percentage of the no cancer group suffering from comorbid health conditions tended to be lower than among cancer survivors. The mean (SD) MCS and PCS scores in the no cancer population were 50.7 (10.8) and 39.0 (12.2), respectively. The mean unadjusted PCS scores for those diagnosed with cancer, except prostate, were all statistically significantly lower than the mean PCS for participants without cancer. The percentage of individuals reporting difficulty completing activities was significantly higher for those with bladder, breast, colorectal, endometrial/uterine, and lung cancer than those without cancer. The prevalence of UI among cancer patients ranged from 37.0% in lung cancer patients to 53.9% in endometrial/uterine; compared to a prevalence of 36.2% in the no cancer population. Prevalence of UI did not significantly vary by time since diagnosis for all cancer types (data not shown).

**Table 1:** **Distribution of demographic and clinic characteristics by cancer type**^**1**^

	**Bladder**	**Breast**	**Colorectal**	**Endometrial/**	**Lung**	**Prostate**	**No cancer**
				**Uterine**			
	**N=749**	**N=2,828**	**N=1,739**	**N=562**	**N=662**	**N=3,258**	**N=319,734**
	**N (%)**	**N (%)**	**N (%)**	**N (%)**	**N (%)**	**N (%)**	**N (%)**
Age							
>75	486 (64.9) **	1,604 (56.7)**	1,140 (65.6)**	352 (62.6)**	338 (51.1)*	1,985 (60.9)**	147,523 (46.1)
Current smoker							
Yes	114 (15.5)**	227 (8.1)**	128 (7.5)**	19 (3.4)**	121 (18.6)**	295 (9.2)**	35,612 (11.3)
Race							
White	648 (88.6)	2,244 (81.6)	1,576 (79.9)	444 (81.6)	524 (81.5)	2,489 (78.7)	279,313 (85.5)
Non- white	83 (11.4)*	507 (18.4)**	338 (20.1)**	100 (18.4)*	119 (18.5)**	673 (21.3)**	45,395 (14.5)
Gender							
Female	225 (30.0)	2,828 (100)	883 (50.8)	562 (100)	343 (51.8)	0 (0)	191,922 (60.0)
Male	524 (70.0)**	0 (0)	856 (49.2)**	0 (0)	319 (48.2)**	3,258 (100)	127,812 (40.0)
Marital status							
Not married	275 (38.8)**	1,540 (57.2)**	743 (44.8)	297 (56.0)**	297 (47.2)	755 (24.5)**	132,154 (43.8)
Education							
> High school	286 (38.8)**	1,053 (37.7)**	608 (35.6)*	214 (38.6)**	227 (35.1)	1,421 (44.4)**	102,928 (32.8)
High blood pressure							
Yes	465 (62.8)	1,797 (64.6)**	1,087 (63.2)	359 (64.5)	388 (59.5)	1,891 (58.7)**	193,321 (61.1)
Stroke							
Yes	87 (11.8)*	218 (7.8)*	193 (11.3)**	40 (7.2)	82 (12.6)**	345 (10.7)**	28,737 (9.1)
Cardio vascular disease							
Yes	339 (45.6)**	833 (29.7)**	605 (35.2)	169 (30.2)	264 (40.4)**	1,136 (35.1)	107,374 (33.8)
Chronic lung disease							
Yes	123 (16.9)*	392 (14.1)	228 (13.4)	59 (10.7)*	290 (44.8)**	446 (13.9)	44,038 (14.0)
Gastro intestinal problems							
Yes	40 (5.5)	153 (5.6)	173 (10.4)**	39 (7.1)*	30 (4.7)	130 (4.1)*	15,847 (5.1)
Diabetes							
Yes	162 (22.1)	567 (20.4)	394 (23.1)**	122 (22.1)	138 (21.1)	664 (20.6)	64,412 (20.4)
Difficulty							
Yes	379 (50.9)**	1,334 (47.5)**	816 (47.6)*	306 (54.8)**	402 (61.4)**	1,415 (43.9)	142,992 (45.0)
Joint pain							
Yes	377 (50.9)	1,603 (57.2)**	827 (48.1)**	319 (57.4)*	311 (47.4)*	1,498 (46.2)**	165,509 (52.1)
UI							
Yes	280 (37.9)	1,296 (46.5)**	635 (37.1)	299 (53.9)**	241 (37.0)	1,416 (44.1)**	113,995 (36.2)
MCS							
Mean (SD)	49.5 (10.9)**	51.1 (10.6)	50.1 (10.7)*	51.5 (10.7)**	46.3 (12.2)**	51.0 (10.6)	50.7 (10.8)
PCS							
Mean (SD)	37.0 (11.6)**	37.3 (12.0)**	37.6 (11.8)**	36.4 (12.1)**	31.7 (11.4)**	39.4 (11.9)*	39.0 (12.2)
Time since cancer diagnosis							
<2years	213 (28.4)	567 (20.0)	392 (22.5)	66 (11.7)	342 (51.7)	716 (22.0)	N/A
2-5years	172 (23.0)	614 (21.7)	403 (23.2)	62 (11.0)	110 (16.6)	811 (24.9)	N/A
≥5years	364 (48.6)	1,647 (58.2)	944 (54.3)	434 (77.2)	210 (31.7)	1,731 (53.1)	N/A

^1^Two-tailed p-value for chi-squared test, comparing distribution of covariate of cancer group to no cancer group where applicable, or t-test for HRQoL scores.

* p <0.05.

** p <0.01.

Among participants with and without cancer who reported UI, the magnitude of the UI problem was further explored (Table 2). 13.1% of non-cancer participants with UI reported that their UI was a “big problem.” The cancer type with the highest percentage reporting that their UI was a “big problem” was endometrial/uterine cancer patients at 20.1%. Cancer patients were in general more likely than non-cancer participants to talk to their physician about urinary leakage, although this was only statistically significantly different for bladder and prostate cancer patients. Approximately a quarter of the non-cancer group who reported having UI received treatment, whereas 37.4% of bladder patients received treatment.

**Table 2:** **Magnitude of UI problem by cancer among those who reported UI**^**1**^

		**Bladder**	**Breast**	**Colorectal**	**Endo/ Uterine**	**Lung**	**Prostate**	**No cancer**
		**N (%)**	**N (%)**	**N (%)**	**N (%)**	**N (%)**	**N (%)**	**N (%)**
		**N=280**	**N=1,296**	**N=635**	**N=299**	**N=241**	**N=1,416**	**N=113,995**
Magnitude of UI problem	Big	50 (13.3)	240 (14.9)	120 (13.9)	60 (20.1)	47 (14.2)	266 (14.5)	20,675 (13.1)
	Small	174 (46.3)	769 (47.9)	380 (44.0)	169 (56.4)	150 (45.3)	839 (45.7)	69,052 (43.9)
	Not a problem	152 (40.4)	597 (37.2)**	364 (42.1)	70 (23.5)	134 (40.5)	731 (39.8)*	67,607 (43.0)
Talked to physician about UI	Yes	175 (54.3)**	514 (36.9)	257 (34.0)	112 (44.4)	110 (37.7)	878 (56.5)**	47,655 (35.4)
Received treatment for UI	Yes	107 (37.4)**	291 (24.9)	135 (21.4)	74 (37.0)	50 (19.2)	477 (34.0)**	27,238 (24.1)

^1^Two-tailed p-value for chi-squared test, comparing distribution of covariate of cancer group to no cancer group.

* p <0.05.

**p<0.01.

After fitting a logistic regression model for cancer and non-cancer groups, factors associated with UI were identified (Table 3). Prevalence Odds Ratios (PORs) and 95% confidence intervals were calculated from logistic models due to the cross-sectional data structure. Being diagnosed with prostate cancer (POR=2.43, 95% CI: 2.25, 2.63) was associated with the largest increase in odds of prevalent UI, as was being diagnosed with bladder (POR=1.25, 95% CI: 1.06, 1.47) and endometrial/uterine (POR=1.31, 95% CI: 1.09, 1.58) cancers; whereas being diagnosed with either colorectal or lung cancer was not associated with an increase in the odds of UI. Older age, being a non-smoker, non-Hispanic white race, female gender, higher education, difficulty completing activities, and the presence of stroke, chronic lung disease, gastrointestinal problems, diabetes, joint pain, and cardiovascular disease were also associated with increased odds of UI. Two of the strongest factors associated with UI were being female (POR (male)=0.42, 95% CI: 0.41, 0.43) and having difficulty completing activities (POR=1.95, 95% CI: 1.91, 1.98). When fitting the same model only among cancer patients, a slight decrease in odds of UI (Table 4; POR=0.88, 95% CI 0.77, 1.01) was observed for cancer survivors between 2-5 years from diagnosis relative to those less than 2 years from diagnosis. A similar small decrease in odds of UI was seen for cancer survivors more than 5 years from their diagnosis (POR=0.94, 95%CI 0.84, 1.05).

**Table 3:** Logistic regression results for associations with UI compared to older Americans without cancer

	**Variable**	**POR**	**95% CI**	**P**
Cancer type^1^	Bladder	1.25	(1.06, 1.47)	0.01
	Breast	1.10	(1.02, 1.20)	0.02
	Colorectal	1.07	(0.95, 1.19)	0.3
	Endometrial/Uterine	1.31	(1.09, 1.58)	0.004
	Lung	0.96	(0.80, 1.14)	0.6
	Prostate	2.43	(2.25, 2.63)	<.0001
Demographics	Age (>75years)	1.20	(1.18, 1.22)	<.0001
	Current Smoker	0.87	(0.85, 0.89)	<.0001
	Race (non-white)	0.69	(0.67, 0.71)	<.0001
	Gender (male)	0.42	(0.41, 0.43)	<.0001
	Marital Status (not married)	1.01	(0.99, 1.03)	0.2
	Education (> high school)	1.33	(1.31, 1.35)	<.0001
Comorbid conditions	High blood pressure	1.02	(1.00, 1.03)	0.06
	Stroke	1.34	(1.31, 1.38)	<.0001
	Chronic lung disease	1.19	(1.16, 1.22)	<.0001
	Gastrointestinal problems	1.40	(1.35, 1.45)	<.0001
	Diabetes	1.15	(1.12, 1.17)	<.0001
	Joint Pain	1.45	(1.42, 1.47)	<.0001
	Cardiovascular disease	1.21	(1.19, 1.23)	<.0001
Activities of daily living	Difficulty	1.95	(1.91, 1.98)	<.0001

^1^Noncancer participants are referent for cancer type.

**Table 4:** Logistic regression results for associations with UI among cancer cases, controlling for time since diagnosis

	**Variable**	**POR**	**95% CI**	**P**
Cancer type^1^	Bladder	1.12	(0.92, 1.37)	0.3
	Breast	1.15	(0.99, 1.34)	0.08
	Colorectal	referent	--	-
	Endometrial/Uterine	1.39	(1.11, 1.74)	0.005
	Lung	0.90	(0.73, 1.12)	0.4
	Prostate	1.95	(1.66, 2.29)	<.0001
Demographics	Age (>75years)	1.12	(1.02, 1.24)	0.01
	Current Smoker	0.98	(0.84, 1.14)	0.8
	Race (non-white)	0.82	(0.73, 0.92)	0.0001
	Gender (male)	0.55	(0.47, 0.66)	<.0001
	Marital Status (not married)	1.03	(0.94, 1.14)	0.5
	Education (> high school)	1.22	(1.11, 1.34)	<.0001
Comorbid conditions	High blood pressure	1.00	(0.91, 1.10)	0.9
	Stroke	1.31	(1.13, 1.53)	0.0005
	Chronic lung disease	1.10	(0.97, 1.25)	0.1
	Gastrointestinal problems	1.38	(1.14, 1.68)	0.001
	Diabetes	1.23	(1.09, 1.37)	0.0004
	Joint Pain	1.66	(1.36, 1.63)	<.0001
	Cardiovascular disease	1.49	(1.00, 1.22)	0.05
Time from cancer to survey	<2years	referent	--	-
	2-5years	0.88	(0.77, 1.01)	0.07
	≥5years	0.94	(0.84, 1.05)	0.3
Activities of daily living	Difficulty	1.66	(1.50, 1.83)	<.0001

^1^Colorectal cancer patients within 2years of treatment are referent group.

Results from linear regression of variables associated with MCS and PCS, are presented in Table 5. UI was significantly associated with a 1.27 point decrease in PCS score (β=−1.27, 95% CI: -1.34, -1.20) and an almost 2-point reduction in MCS (β=−1.75, 95% CI: -1.83, -1.68), after adjusting for cancer type, demographics, and comorbidities; although these differences may not be clinically relevant. All of the cancer types had negative associations with PCS; with lung cancer being associated with the largest detrimental effect on PCS score (β=−4.25, 95% CI: -4.95, -3.54) relative to the non-cancer group. Of the cancer types, only lung cancer had a significant negative impact on MCS scores with a decrease on average of 2.77 points (β=−2.77, 95% CI: -3.57, -1.97) relative to non-cancer participants. Difficulty completing activities of daily living resulted in the largest decrease in both PCS (β=−12.95, 95% CI: -13.02, -12.88) and MCS (β=−5.57, 95% CI: -5.65, -5.49) compared to those who did not report any difficulty with activities of daily living.

**Table 5:** Linear regression associations with PCS and MCS compared to older Americans without cancer

	**Variable**	**PCS**			**MCS**		
		**β**	**95% CI**	**P**	**Β**	**95% CI**	**P**
Urinary Incontinence	UI	−1.27	(−1.34, -1.20)	<.0001	−1.75	(−1.83, -1.68)	<.0001
Cancer type^1^	Bladder	−1.12	(−1.79, -0.46)	0.0009	−0.59	(−1.35, 0.16)	0.1
	Breast	−0.72	(−1.06, -0.38)	<.0001	0.52	(0.13, 0.91)	0.009
	Colorectal	−0.85	(−1.29, -0.41)	<0.001	−0.28	(−0.78, 0.22)	0.3
	Endo/ Uterine	−0.39	(−1.16, 0.37)	0.3	1.62	(0.75, 2.48)	0.003
	Lung	−4.25	(−4.95, -3.54)	<.0001	−2.77	(−3.57, -1.97)	<.0001
	Prostate	−0.16	(−0.48, 0.16)	0.3	0.17	(−0.19, 0.53)	0.4
Demo-graphics	Age (>75years)	−1.50	(−1.57, -1.43)	<.0001	−0.06	(−0.13, 0.02)	0.1
	Current Smoker	−0.82	(−0.93, -0.72)	<.0001	−2.30	(−2.41, -2.18)	<.0001
	Race (non-white)	−1.09	(−1.18, -1.00)	<.0001	−2.09	(−2.20, -1.98)	<.0001
	Gender (male)	0.76	(0.69, 0.83)	<.0001	−0.35	(−0.43, -0.27)	<.0001
	Marital Status (not married)	−0.02	(−0.08, 0.06)	0.6	−1.03	(−1.10, -0.95)	<.0001
	Education (> high school)	1.62	(1.55, 1.69)	<.0001	1.92	(1.84, 1.99)	<.0001
Comorbid conditions	High blood pressure	−0.94	(−1.00, -0.87)	<.0001	0.02	(−0.06, -0.09)	0.7
	Stroke	−2.17	(−2.28, -2.05)	<.0001	−2.17	(−2.30, -2.04)	<.0001
	Chronic lung disease	−3.52	(−3.62, -3.43)	<.0001	−1.55	(−1.65, -1.44)	<.0001
	Gastro intestinal problems	−1.93	(−2.07, -1.78)	<.0001	−3.40	(−3.57, -3.24)	<.0001
	Diabetes	−1.53	(−1.61, -1.45)	<.0001	−1.04	(−1.13, -0.94)	<.0001
	Joint pain	−3.50	(−3.57, -3.43)	<.0001	−0.57	(−0.65, -0.50)	<.0001
	Cardiovas cular disease	−2.57	(−2.64, -2.50)	<.0001	−1.03	(−1.11, -0.95)	<.0001
Activities of daily living	Difficulty	−12.95	(−13.02, -12.88)	<.0001	−5.57	(−5.65, -5.49)	<.0001

^1^Noncancer participants are referent for cancer type.

## Discussion

To our knowledge, this is the first study to assess the prevalence of UI, identify factors associated with UI, and determine the HRQOL impact of UI in older Americans with and without cancer. Among this population of Medicare beneficiaries age 65 years or older, UI was highly prevalent with over one-third of non-cancer participants reporting UI. A higher prevalence of UI was observed for all cancer types included in this analysis compared to the non-cancer population, although the cancer population was relatively older. Bladder, endometrial/uterine, and prostate cancer diagnoses had the strongest associations with UI. UI was also associated with decreased PCS and MCS, after controlling for being diagnosed with cancer, demographic, and comorbid conditions.

There was a suggestion of a decrease in odds of UI with increasing time from diagnosis among cancer survivors, relative to the colorectal cancer group which had a similar prevalence of UI as the non-cancer Medicare beneficiaries. This may be due to recovery of urinary control after cancer treatment [23] or adaptation after cancer treatment.

The symptoms and treatments for pelvic cancers, such as bladder, endometrial/uterine, and prostate cancer, are known to be associated with an increased risk of urinary incontinence [24,25]. However, the prevalence of UI in cancer survivors on a population-level has not been previously examined. Previous research has suggested that urinary incontinence symptoms may be relatively common after radical hysterectomy [16-18]. Previously it has been suggested that women who undergo hysterectomy for treatment of gynecological cancers may have decreased quality of life, including decreased sexual function [26,27], which could explain the higher proportion reporting UI as a serious concern. In this current study, women with endometrial/uterine cancer had the highest self-reported burden of symptoms, labeling their UI a ‘big problem’

The prevalence of UI for individuals without cancer was comparable to estimates reported previously for females, although higher than the general population prevalence estimates previously reported for men [5,6]. However, this is an older population so the prior data with slightly lower rates are not necessarily applicable to Medicare beneficiaries. Our method of using self-reported UI is possibly a more accurate reflection of the prevalence of UI symptoms than a clinical measurement as UI is often underdiagnosed [28]. This is evident given that only 35% to 57% of the SEER-MHOS participants who self-reported UI indicated that they had spoken to their doctor about their UI symptoms.

HRQOL measures were negatively associated with UI, history of certain cancers, demographics, and comorbid conditions. UI, regardless of cancer diagnosis and other covariates in the models, was independently significantly associated with decreases in both MCS and PCS, especially larger decreases observed for mental health. This suggests that UI is an important factor in the lives and health of Medicare beneficiaries whether or not they have been diagnosed with cancer.

This study would have benefited from being able to assess the impact of cancer treatment, particularly surgery, on the prevalence of UI. However, due to large amounts of missing treatment data in SEER, this was not possible. Additionally, this analysis was cross-sectional and therefore is limited in drawing any conclusions regarding temporality between the cancer diagnosis, UI, and HRQOL. Although there were a wide range of variables collected from the MHOS, there were unmeasured risk factors for UI that we could not address in this analysis including reproductive history [29,30] and other urological conditions that could lead to UI.

A strength of this investigation is the large sample size provide by the linked SEER-MHOS database. The ability to compare across different cancer types and also between those with and without cancer is a major advantage of this analysis. In addition, various demographic and comorbid conditions that had an impact on HRQOL scores and prevalence of UI were accounted for in this analysis.

## Conclusions

With a growing, vulnerable population of cancer survivors [20], further investigation of health problems survivors are more likely to face is crucial. In particular, identifying comorbid conditions, such as UI, that negatively impact HRQOL has public health importance, as cancer survivors already demonstrate decrements in HRQOL scores [25]. Multiple treatment options for persistent UI, particularly among cancer survivors who had surgery such as prostatectomy and hysterectomy, have been shown to be effective in lessening symptoms of UI [31-34]. In this study, gastrointestinal problems, chronic lung disease and joint pain were strongly associated with decreased HRQOL scores. The understanding of UI prevalence and HRQOL impact of UI among cancer survivors may assist in focusing survivorship care efforts to improve the long-term health and HRQOL of this patient population.

## Competing interests

The authors have no conflicts of interest or financial disclosures.

## Authors’ contributions

AW and DI conceived and designed the analysis with contributions from BR, AS and RC. AW and DI drafted the manuscript. AW carried out statistical analysis. All authors contributed to the interpretation of the data, revisions of the manuscript and read and approved the final manuscript.

## Pre-publication history

The pre-publication history for this paper can be accessed here:

http://www.biomedcentral.com/1471-2407/13/377/prepub
